# Diagnosis of sepsis with inflammatory biomarkers, cytokines, endothelial functional markers from SIRS patients

**DOI:** 10.1097/MD.0000000000028681

**Published:** 2022-02-18

**Authors:** Mingming Xue, Feixiang Xu, Yilin Yang, Zhengang Tao, Yumei Chen, Sheng Wang, Jun Yin, Min Min, Dongwei Shi, Chenling Yao, Zhenju Song

**Affiliations:** Department of Emergency Medicine, Zhongshan Hospital, Fudan University, Shanghai, China.

**Keywords:** cytokines, endothelial functional markers, inflammatory biomarkers, sepsis, SIRS

## Abstract

**Background:**

Sepsis is a life-threatening illness with a challenging diagnosis. Rapid detection is the key to successful treatment of sepsis. To investigate diagnostic value, the plasma protein profiles of inflammatory biomarkers, cytokines, and endothelial functional markers were compared between healthy controls, SIRS, and septic patients.

**Methods:**

The plasma protein profiles were performed by Luminex Assay in a cohort of 50 SIRS patients, 82 septic patients and 25 healthy controls. Fourteen plasma proteins were analyzed in the same cohort: IL-1β, IL-6, IL-8, IL-10, CCL-2, VEGF, VEGF-C, VEGFR2, CD62E, CD62P, MFG-E8, ICAM-1, TFPI, Urokinase.

**Result:**

IL-2R, IL-6, IL-8, IL-10, CCL-2, ICAM-1, and Urokinase were significantly higher in sepsis patients than SIRS patients. VEGF, IL-1β, CD62E, CD62P, MFG-E8, and TFPI have no statistical difference. VEGF-C, VEGFR2 were significantly different in SIRS patients than sepsis patients. Urokinase, ICAM-1, and VEGFR2 were significantly different between sepsis group and SIRS group. The AUCs of Urokinase, ICAM-1, and VEGFR2 and the combination for the diagnosis of sepsis were 0.650, 0.688, 0.643, and 0.741, respectively.

**Conclusions:**

Most patients have the higher level of several cytokines and developed endothelial cell injury in the initial phase of sepsis, Urokinase, ICAM-1, and VEGFR2 may be useful to evaluate severity and prognosis of sepsis patients.

## 1 Introduction

Sepsis as a systemic inflammatory response syndrome (SIRS) caused by an infection can cause damage to the body tissues and organs themselves and can even be life-threatening.^[^1^–^3^]^ It is difficult to recognize patients with sepsis because without a specific clinical signs.^[^4^,^^5^^]^ Despite the great improvement in detect this disease,^[^6^–^8^]^ septic patients are a heterogeneous group and their condition has been very difficult to recognize especially in the early stages.^[^9^,^^10^^]^

The definition of sepsis which involves SIRS plus proof or suspicion of infection, could be very broad and it includes a large number of patients who do not develop sepsis. At the same time, the transition from SIRS to sepsis and septic shock develops over time differently in various patients so that even the progression of serious disease could be unrecognized until it reaches the late stage. Additionally, early clinical signs of sepsis are variable and nonspecific. Therefore, considering these limitations in diagnosis of sepsis and the importance of reducing mortality and cost of care, the evaluation system of sepsis should be developed. Early sepsis is easily misdiagnosed as SIRS, leading to delayed treatment. Therefore, more specific markers are required for the diagnosis of sepsis.

The aim of this study was to investigate the importance and difference of several biomarkers to distinguish patients with sepsis from those with infectious SIRS.

## 2 Methods

### 2.1 Patients and study design

A retrospective study was carried out in the emergency department of Zhongshan Hospital, Fudan University, Shanghai, China. Fifty SIRS patients, 82 septic patients, and 25 healthy controls on admission from October 2019 to September 2020 were enrolled in this study. According to the ACCP/SCCM (American College of Chest Physicians/Society of Critical Care Medicine), in all patients who met two or more criteria for SIRS (body temperature body temperature >38 or <36°C, heart rate >90 beats/min, respiratory rate >20 breath/min or pCO_2_ < 4.3 kPa, white blood cell count >12.0 × 10^9^/L or <4.0 × 109/L, or >10% immature forms) were enrolled. The diagnosis of sepsis referred to The Third International Consensus Definitions for Sepsis and Septic Shock (Sepsis-3), namely, suspected infection with a Sequential Organ Failure Assessment (SOFA) score ≥2.

The exclusion criteria were as follows:

1. age < 18 years;
2. immune system disease;
3. malignancy;
4. hematological disease;
5. thrombotic disease;
6. chronic liver disease; and
7. chronic renal failure requiring hemodialysis.

The study was approved by the Institutional Research Ethics Committee of Zhongshan Hospital, Fudan University (No: 2006-23). All of the cases and controls were enrolled consecutively. Informed consent was waived because of the retrospective nature of this study.

### 2.2 Severity and outcome assessment

The Acute Physiology and Chronic Health Evaluation (APACHE) II score was assessed at the enrollment of the patients with sepsis. The APACHE II score is designed to assess the severity of critically ill patients based on physiologic measurements, age and previous health status and is used for the prediction of outcome in critically ill patients. The Sequential Organ Failure Assessment (SOFA) score was assessed at the same time points that blood samples were taken. The SOFA is a scoring system composed of six organ systems (comprising the respiratory, coagulation, hepatic, cardiovascular, renal, and nervous systems) and can be used for the evaluation of organ failure and prognosis.

### 2.3 Data collection

Patient baseline data, including age, sex, site of infection, APACHE II, and SOFA scores, were collected from the Electronic Medical Record System (EMRS). Underlying medical history was also obtained, including ischemic heart disease, chronic heart failure, chronic obstructive pulmonary disease, cerebrovascular accident, and diabetes mellitus. The levels of coagulation indices (prothrombin time [PT], thrombin time [TT], activated partial thromboplastin time [APTT], fibrinogen [FIB] and D-dimer, platelet count [PLT], and lactate were collected. Blood coagulation, including those for PT, TT, APTT, and FIB, was assayed using the CS-2100i automatic coagulation analyzer (Sysmex).

### 2.4 Biomarker measurement

After admission to the emergency department, 2 to 3 mL venous blood was drawn from septic and SIRS control patients upon enrollment and added to a tube containing sodium citrate (BD Vacutainer with sodium citrate anticoagulant, UK). The blood samples were centrifuged at 3000 rpm at 4°C for 10 minutes, and then the supernatant was stored at −80°C for use. The frozen plasma samples were thawed just before use, by immersion in a water bath at 37°C for 5 minutes.

Preparation of antibody conjugated Luminex^TM^ beads. Coupling Luminex MagPlex-COOH beads (Bio-Rad; Hercules, CA) to monoclonal antibodies was based on the procedures outlined in the X-MAP Cookbook. All primary antibodies that contained amine containing additives or preservations were cleaned using Micro Bio-Spin 6 Tris chromatography columns (Bio-Rad; Hercules, CA) according to the manufacture's instructions. Conjugation of antibodies to Luminex^TM^ MagPlex-COOH beads was performed using the Bio-Rad bead making kit, and conjugated beads were quantified using a TC20 cell counter.

### 2.5 Statistical analysis

Normally distributed continuous variables were expressed as the means ± standard deviations, and abnormally distributed continuous variables were expressed as medians (25th and 75th quartiles). Student's *t* test or one-way analysis of variance was used to compare normally distributed continuous variables. Kruskal–Wallis one-way analysis or Mann–Whitney *U* test was utilized to compare nonnormally distributed continuous variables. Categorical data were expressed as numbers (percentages) and compared with Pearson's chi-square test or Fisher's exact test when appropriate. Receiver operating characteristic (ROC) curves were constructed, and the areas under the ROC curves (AUCs) were determined. A multivariate logistic regression model based on a forward stepwise method was used to identify the abnormal parameters. The hazard ratios with 95% confidence intervals (CIs) to show markers of all-cause mortality. All statistical analyses were two-tailed, and the significance level was set to *P* < .05. Statistical analyses were performed using SPSS (version 22.0, SPSS, Chicago, IL).

## 3 Results

### 3.1 Demographic characteristics of the study population

One hundred thirty-two SIRS patients and 25 healthy controls were consecutively enrolled in this study, 82 of SIRS patients were diagnosed with sepsis. The baseline characteristics of the study population were shown in Table 1. Of the patients with sepsis, 33 were men and 49 were women, and the mortality rate of these patient was 30.5%. There was no significant difference in age and sex between control, SIRS and septic patients. The median of APACHE II and SOFA scores had significantly difference between three groups. The most common sources of infection in sepsis group were respiratory system (28/82, 34.1%), urinary tract infection (22/82, 26.8%), liver abscess (18/82, 22.0%), abdominal infection (12/82, 14.6%), soft tissue infection (2/82, 2.4%), central nervous system infection (4/82, 4.9%), and other (2/82, 2.4%). Etiology of infections in sepsis group were: Gram-negative bacterial infections in 21(26%), Gram-positive bacterial infections in 45 (54.9%), fungal/viral infections in 11 (13.4%) and unknown infection in 5 (6.1%).

**Table 1:** Baseline characteristic of sepsis and control patients.

Variable	Healthy control (N = 25)	SIRS (N = 50)	Sepsis (N = 82)	*P*
Age (yr)	58.0 ± 12.9	59.2 ± 16.9	61.0 ± 17.0	.769
Male	17	58	33	.478
Temp (°C)	36.5 (36, 36.6)	37.9 (37,38.6)	37.8 (37.1, 39)	<.001
SOFA score	0 (0, 0)	2 (0, 4)	5 (3, 8)	<.001
APACHEII score	2.5 (0, 3)	9.0 (5.8, 12.3)	13.5 (9.0, 19.3)	<.001
Mortality rate	0	0	25 (30.5%)	<.001
Complication				
Diabetes	2	17	16	.065
Hypertention	5	23	34	.425
Coronary artery disease	3	3	12	.061
Chronic liver disease	0	1	2	.078
Chronic renal disease	0	3	4	.095
Chronic pulmonary disease	0	3	5	.069
Cause of infection				
Pneumonia	0	19	28 (34.1%)	
Liver abscess	0	11	18 (22.0%)	
Urinary tract infection	0	12	22 (26.8%)	
Abdominal infection	0	6	12 (14.6%)	
Soft tissue infection	0	2	2 (2.4%)	
Central nervous system infection	0	0	4 (4.9%)	
Other	0	0	2 (2.4%)	
Infection etiology				
Gram positive	0	12	21 (26%)	
Gram negtive	0	30	45 (54.9%)	
Fungal/viral infection	0	5	11 (13.4%)	
Unknown	0	3	5 (6.1%)	

### 3.2 Laboratory measurements of septic patients and SIRS patients at enrollment

We analyzed the laboratory findings between healthy control, sepsis and SIRS patients. We found that sepsis patients had significantly higher levels of WBC, neutrophil percent (N%), C-reactive (CRP), procalcitonin (PCT), lactate, and lymphocyte percent (L%) (Table 2). The count of platelets were significantly decreased in the sepsis group compared with control and SIRS group. The level of Hb in septic patients was lower than SRIS group, but there was not statistically significant. The levels of PT, APTT, and INR were significantly increased in sepsis patients compared with SIRS patients throughout the study period. There was no significantly difference in the level of TT, Fib, and D-Dimer. The level of LDH was significantly higher in sepsis group. The level of BUN was significantly higher in sepsis group, but there were no significance between creatinine and glomerular filtration rate (GFR). The level of proBNP was significantly higher in sepsis group. The level of cTnT was no significance between two groups. The level of CK-MM was significantly higher in sepsis group, but there were no significance between CK, CK-MB, and myoglobin.

**Table 2:** Characteristics of laboratory values and univariate analysis between SIRS and Sepsis groups.

	Healthy control (N = 25)	SIRS (N = 50)	Sepsis (N = 82)	*P*
WBC (∗10^9^/L)	5.58 ± 0.89	7.95 ± 3.88	13.35 ± 7.81	<.001
Neutrophil percentage	52.37 ± 6.29	69.14 ± 13.42	82.76 ± 12.75	<.001
CRP (mg/L)	0.73 ± 0.87	47.24 ± 65.75	111.22 ± 107.89	<.001
PCT (ng/mL)	0.3 ± 0.12	5.27 ± 16.33	18.93 ± 33.04	.002
Lymphocyte percentage	38.41 ± 5.70	19.39 ± 10.76	10.62 ± 11.35	<.001
Hb (g/L)	144 ± 10.85	113.44 ± 23.04	106.96 ± 23.01	.119
PLT(∗10^9^/L)	223.58 ± 51.96	268.78 ± 130.25	150.06 ± 108.37	<.001
Lactate (mmol/L)	0.52 ± 0.20	1.51 ± 0.37	3.05 ± 2.68	<.001
PT (s)	9.55 ± 1.09	13.39 ± 2.35	17.53 ± 13.46	.009
INR	0.98 ± 0.08	1.20 ± 0.22	1.78 ± 2.20	.023
TT (s)	15.34 ± 0.77	16.17 ± 3.47	18.61 ± 16.79	.355
APTT (s)	23.78 ± 1.24	29.18 ± 3.64	32.81 ± 9.98	.005
Fib (mg/dL)	227.08 ± 35.55	390.76 ± 119.62	445.35 ± 198.77	.062
D-Dimer (mg/L)	0.46 ± 0.12	17.94 ± 78.04	8.08 ± 8.73	.43
TB ( μmol/L)	14.57 ± 4.69	11.84 ± 10.67	28.27 ± 45.15	.003
CB ( μmol/L)	2.91 ± 0.81	5.27 ± 6.76	16.92 ± 36.05	.007
ALT (U/L)	27.58 ± 10.04	231.87 ± 1228.15	195.49 ± 506.66	.818
AST (U/L)	27.42 ± 5.33	362.16 ± 2231.72	245.73 ± 769.7	.675
LDH (U/L)	204.25 ± 27.60	259.81 ± 132.04	564.14 ± 409.49	.001
ALP (U/L)	72.08 ± 13.71	102.64 ± 56.12	114.42 ± 122.64	.544
Creatinine (μmol/L)	69 ± 11.51	90.6 ± 96.92	119.78 ± 107.69	.126
Urea nitrogen (mmol/L)	4.81 ± 1.02	6.88 ± 3.54	11.76 ± 7.68	<.001
GFR (mL/min/1.73 m^2^)	98.28 ± 10.22	87.16 ± 29.65	75.05 ± 37.33	.064
cTnT (ng/mL)	0.019 ± 0.022	0.34 ± 1.80	0.11 ± 0.22	.396
proBNP (pg/mL)	81.5 ± 28.64	704.51 ± 1146.38	4111.63 ± 6469.18	<.001
CK (U/L)	76.17 ± 22.72	63.03 ± 75.97	1066.30 ± 5414.46	.263
CK-MB (U/L)	15.7 ± 1.85	12.17 ± 4.66	92.39 ± 581.51	.411
CK-MM (U/L)	77.63 ± 26.52	52.19 ± 75.71	494.24 ± 1207.56	.003

### 3.3 Plasma levels of cytokines and endothelial functional markers at enrollment

**Figure 1:**
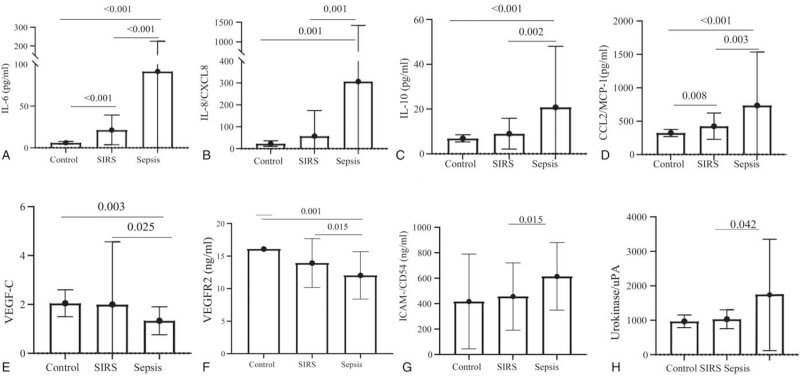
Serum levels of IL-6, IL-8, IL-10, CCL2, VEGF-C, VEGFR2, ICAM-1, and Urokinase/uPA at enrollment between healthy control, SIRS and sepsis groups.

The levels of IL-2R, IL-6, IL-8,IL-10, and MCP-1 were significantly increased in sepsis patients compared with SIRS patients throughout the study period in Table 3. There was no significantly difference in the level of IL-1β. The levels of VEGF-C and VEGFR-2 were decreased in sepsis patients compared with SIRS patients. Figure S1 shows that the level of VEGF has no significantly difference (Figure S1, Supplemental Digital Content, which demonstrates the plasma levels of VEGF at enrollment between different groups). The level of ICAM-1was significantly increased in sepsis patients compared with SIRS patients throughout the study period. The levels of u-Plasminogen Activator were significantly increased in sepsis patients compared with SIRS patients. There were no significantly difference in the levels of E-Selection, P-Selection, MFG-E8, and TFPI (Fig. 1).

**Table 3:** Characteristics of cytokines and endothelial functional markers between different groups.

	Healthy control (N = 25)	SIRS (N = 50)	Sepsis (N = 82)	*P*
TNFa	5.61 ± 1.61	26.04 ± 27.6	32.61 ± 81.78	.664
IL-1β (pg/mL)	12.41 ± 7.12	30.55 ± 42.61	48.37 ± 95.71	.216
IL-2R (pg/mL)	419.58 ± 132.00	980.53 ± 409.61	1407.47 ± 1102.19	.006
IL-6 (pg/mL)	6.04 ± 1.51	21.33 ± 17.73	321.78 ± 1179.01	.024
CXCL8/IL-8 (pg/mL)	23.04 ± 12.78	57.54 ± 116.9	306.62 ± 1117.76	.049
IL-10 (pg/mL)	6.87 ± 1.59	8.97 ± 6.84	20.76 ± 27.30	<.001
CCL-2/MCP-1 (pg/mL)	323.91 ± 54.02	423.4 ± 196.79	735.54 ± 801.27	.001
VEGF (pg/mL)	79.96 ± 48.38	232.31 ± 167.00	228.31 ± 265.23	.924
VEGF-C (ng/mL)	2.05 ± 0.55	2.00 ± 2.57	1.33 ± 0.57	.025
VEGFR2/kdr/Flk-1 (ng/mL)	16.08 ± 2.84	13.92 ± 3.75	12.02 ± 3.64	.005
CD62E/E-Selection (ng/mL)	29.34 ± 7.42	69.13 ± 36.5	77.08 ± 51.34	.302
CD62P/P-Selection (ng/mL)	37.46 ± 8.09	41.21 ± 16.26	40.52 ± 15.14	.805
MFG-E8 (ng/mL)	1.02 ± 0.39	1.62 ± 1.12	1.77 ± 1.09	.443
ICAM-1/CD54 (ng/mL)	416.66 ± 372.98	456.08 ± 264.56	613.85 ± 265.49	.001
TFPI (ng/mL)	16.84 ± 6.80	17.03 ± 7.42	20.25 ± 22.4	.233
Urokinase/u-Plasminogen Activator (uPA) (pg/mL)	968.31 ± 183.75	1030.2 ± 275.22	1432.08 ± 1922.42	.045

### 3.4 Plasma levels of Urokinase, ICAM-1, VEGFR2, and combined for sepsis diagnosis

**Figure 2:**
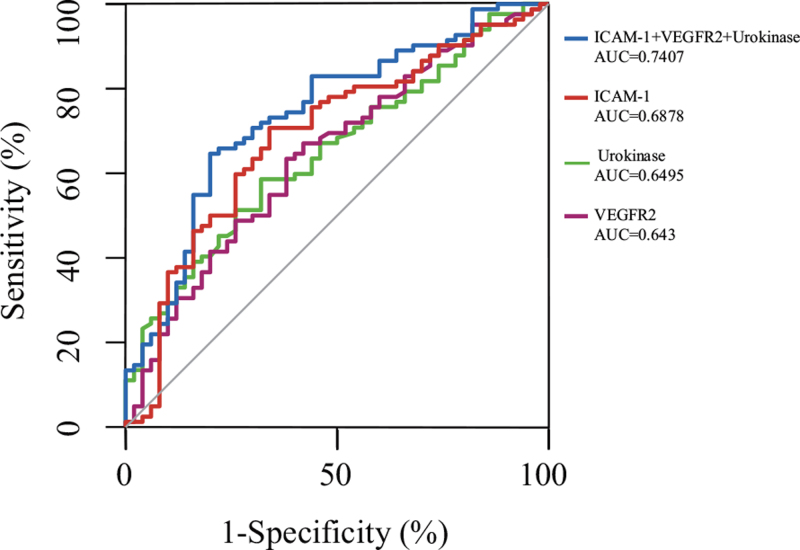
Serum levels of ICAM-1, VEGFR2, and Urokinase for sepsis diagnosis. Receiver operating characteristic (ROC) curves of ICAM-1, VEGFR2, and Urokinase levels for diagnosing sepsis in SIRS patients. The area under the ROC curve (AUC) of ICAM-1 was 0.7407, that of Urokinase was 0.6878 and that of VEGFR-2 was 0.643.

We performed multivariate logistic regression analysis among SIRS and sepsis patient, found that the levels of Urokinase, ICAM-1, and VEGFR2 were independent predictors for diagnosis after multivatiate analysis (Tables 4 and 5). The ORs of the Urokinase, ICAM-1, and VEGFR2 were 1.001 (1.000–1.003, *P* = .038), 1.002 (1.001–1.003, *P* = .045), 0.857 (0.768–0.956, *P* = .006), respectively. The evaluation of each marker to diagnosis sepsis was carried out via ROC analysis. ROC curve analysis was used to evaluate the value of different biomarkers to identify the disease status. The AUCs of Urokinase, ICAM-1, VEGF-C, and combined scores were 0.65, 0.688, 0.345, and 0.745, respectively. These values exceeded those for the other markers (Fig. 2).

**Table 4:** Urivariate analysis of cytokines, Urokinase, ICAM-1, VEGF-C for predict sepsis.

Variables	OR (95% CI)	*P*
IL-6	1.024 (1.006, 1.043)	.011
IL-10	1.077 (1.016, 1.142)	.013
CCL2/MCP-1	1.001 (1, 1.002)	.02
IL-2	1.003 (1.002, 1.004)	.021
VEGF	1 (0.998, 1.001)	.923
VEGF-C	0.436 (0.239, 0.796)	.007
VEGFR2	0.868 (0.785, 0.961)	.006
ICAM-1	1.002 (1.001, 1.004)	.002
Urokinase	1.002 (1.001, 1.003)	.004
TFPI	1.012 (0.987, 1.037)	.34

**Table 5:** Multiple factor analysis of Urokinase, ICAM-1, VEGFR2 for predict sepsis adjusted by age and sex.

Variables	OR (95% CI)	*P*
VEGFR2	0.857 (0.768, 0.956)	.006
ICAM-1	1.002 (1.001, 1.003)	.045
Urokinase	1.001 (1, 1.003)	.038
Combined	1.012 (0.987, 1.037)	.994

## 4 Discussion

The ability to diagnose for sepsis is vitally important for patients’ outcomes. In the current study, the results indicated that:

1. Compared healthy controls and SIRS patients, septic patients had higher body temperatures, SOFA and APACHEII scores, and higher mortality.
2. We found that sepsis patients had significantly higher levels of WBC, Neutrophil percent, C-reactive (CRP), procalcitonin (PCT), lactate, lymphocyte percent, LDH, BUN, and proBNP. The count of platelets were significantly decreased in the sepsis group. The cytokine levels of IL-2R, IL-6, IL-8, IL-10, and MCP-1 were significantly increased in sepsis patients compared with SIRS patients.
3. The levels of VEGF-C and VEGFR-2 were decreased in sepsis patients compared with SIRS patients. The level of ICAM-1 and u-Plasminogen Activator was significantly increased in sepsis patients compared with SIRS patients throughout the study period.
4. The AUCs of Urokinase, ICAM-1, VEGFR2, and combined scores were 0.650, 0.688, 0.643, and 0.741, respectively, indicating that Urokinase, ICAM-1, VEGFR2 could be the helpful factors for diagnosis in patients with sepsis.

It is increasingly recognized that sepsis therapy should not be limited to antimicrobial treatment. Instead, great health benefits may be realized by correcting the dysregulated immune response which underlies septic shock pathobiology.^[11]^ Endothelial inflammation is a hallmark of sepsis^[^12^–^14^]^ and leads to shock and MODS.^[^15^,^^16^^]^ Several cytokines produced by activated endothelial have effects on the endothelia themselves and immune cells that interact with them: IL-6 has a role in angiogenesis.^[^17^–^20^]^ Endothelial activation induces expression of ICAM-1, which is involved in sepsis pathogenesis and is markers of end-organ failure, morbidity, and mortality in severe sepsis.^[21]^

It is well known that endothelial activation is crucial in sepsis. During inflammatory states, ICAM-1 was a vascular endothelial surface receptor that allows for leukocytes and other inflammatory cells to bind and translocate into local tissue. Up-to-date, many studies showed that its level is increased in patients with sepsis. Our study indicated that the level of ICAM-1 and u-Plasminogen Activator was significantly increased in sepsis patients.Bavunoglu et al found that ICAM-1 had a high sensitivity (99%) and specificity (99%) for detecting sepsis.^[22]^ Furthermore, multiple mouse models have shown that ICAM-1 knockout mice with severe forms of sepsis have lower mortality rates. A study has demonstrated that ICAM-1 level in the sepsis and the SIRS groups was significantly higher than that in the control group, and it is also higher in the sepsis group than in the SIRS group.^[23]^ Another study also confirmed that high levels of serum ICAM-1 was associated with the development of MOF.^[22]^ Obregon et al provides an interesting insight that ICAM-1 seem to crucial in the pathogenesis and may potentially work as a complementary tool for the physician in the diagnosis of sepsis.^[24]^

In this study, we found the levels of VEGF-C and VEGFR2 were decreased in sepsis patients compared with SIRS patients, and VEGFR2 has diagnostic value in sepsis patients. Vascular endothelial growth factor (VEGF) is crucial for vascular development and stimulate the formation of blood vessels. The over-production of VEGF is associated with acute inflammation. Two receptor tyrosine kinase (RTK) receptors have been identified for VEGF (VEGFR1 and VEGFR2).^[25]^ VEGFR2 is regarded as the main signalling receptor for angiogenesis, proliferation, and permeability.^[26]^ VEGFR2 knockout mice fail to develop blood islands or organised blood vessels resulting in early death.^[27]^ VEGFR2 also has a prosurvival function with anti-apoptotic effects on human umbilical venous endothelial cells (HUVECs).^[28]^ The VEGF family is involved in vascular permeability regulation in sepsis, via yet incompletely understood mechanisms.^[29]^ Aslan et al have shown that after LPS injection in mouse, VEGFR2 expression declined sharply in the lung.^[30]^ Our study confirmed that the plasma level of VEGFR2 decreases associated with severity of infection.

The plasminogen activator (PA) system, an essential system in cell differentiation, migration and reconstruction, mainly contains urokinase-type plasminogen activator (uPA), uPA receptor (uPAR), tissue type plasminogen activator (tPA), plasminogen activator inhibitor-1 (PAI-1), and PAI-2^[31]^ uPA commonly is expressed in neutrophils, monocytes, macrophages, and activated T cells, and is reported to be capable of regulating inflammation, immune responses, and human endothelial cell functions in cancers and several inflammatory diseases.^[^32^–^34^]^ Several studies also report that uPA is beneficial in inflammation by activating T cells and may act as an antibiotic agent in mice model infected with *staphylococcus aureus*.^[^32^–^36^]^ Nonetheless, very limited studies report the impact of uPA in sepsis. Our study confirmed that the plasma levels of u-Plasminogen Activator (uPA) were significantly increased in sepsis patients compared with SIRS patients, and uPA has diagnostic value in sepsis patients. The study by Yang and colleagues examined 81 patients with septic shock due to pneumonia, along with 20 patients with pneumonia without organ dysfunction. Their major findings were that circulating level of urokinase-type plasminogen activator (uPA) was associated with organ dysfunction and mortality, whereas vascular endothelial cell growth factor (VEGF) levels had no such predictive power.^[37]^

Our study had several limitations. First, this study was a single-center, retrospective and observational study, which may increase risk for misclassification biases and confounding. Second, we diminished the effect of confounding actions by clinicians. Third, this study was not a randomized controlled trial, and multiple unmeasured variables might account for the outcome difference observed in the study. Fourth, power analysis indicated that the analyses for the patients enrolled did not have enough statistical power. Thus, further studies with large sample size are needed.

## 5 Conclusions

In conclusion, there are many changes of clinical findings, laboratory parameters and cytokines during sepsis. We found that the endothelial functional markers of Urokinase, ICAM-1 and VEGFR2 were the potential diagnostic tools in patients with suspected sepsis. Monitoring of these markers could raise the possibility to distinguish between patients with sepsis and those with infectious SIRS, leading to early intervention for patients with sepsis.

## Author contributions

**Conceptualization:** Mingming Xue.

**Data curation:** Feixiang Xu, Mian Shao, Chenling Yao.

**Formal analysis:** Jun Yin, Min Min.

**Methodology**: Yilin Yang, Zhenju Song

**Resources:** Chaoyang Tong.

**Software:** Yilin Yang, Zhengang Tao, Yumei Chen, Sheng Wang.

**Supervision:** Dongwei Shi, Chenling Yao.

**Validation:** Chaoyang Tong.

**Visualization:** Zhenju Song.

**Writing – original draft:** Mingming Xue.

**Writing – review & editing:** Chaoyang Tong.

## Supplementary Material

Supplemental Digital Content

## References

[1] Singer M Deutschman CS Seymour CW. The third international consensus definitions for sepsis and septic shock (Sepsis-3). JAMA 2016;315:801–10. 26903338 10.1001/jama.2016.0287 PMC4968574

[2] Stephen AH Montoya RL Aluisio AR. Sepsis and septic shock in low- and middle-income countries. Surg Infect (Larchmt) 2020;21:571–8. 32401160 10.1089/sur.2020.047

[3] Salomão R Ferreira BL Salomão MC. Sepsis: evolving concepts and challenges. Braz J Med Biol Res 2019;52:e8595. 30994733 10.1590/1414-431X20198595 PMC6472937

[4] Dellinger RP Levy MM Rhodes A. Surviving sepsis campaign guidelines committee including the pediatric subgroup. Surviving Sepsis Campaign: international guidelines for management of severe sepsis and septic shock, 2012. Intensive Care Med 2013;39:165–228. 23361625 10.1007/s00134-012-2769-8 PMC7095153

[5] Mirijello A Tosoni A On behalf of The Internal Medicine Sepsis Study Group. New strategies for treatment of sepsis. Medicina (Kaunas) 2020;56:527. 10.3390/medicina56100527 PMC7599752 33050538

[6] Biomarker Insights London, England: SAGE Publications Sage UK, Biron BM Ayala A Lomas-Neira JL. Biomarkers for Sepsis: What Is and What Might Be?. Vol 10s4. 2015;BMI.S29519. 10.4137/BMI.S29519 PMC4571989 26417200

[7] Zhang Z Hong Y. Development of a novel score for the prediction of hospital mortality in patients with severe sepsis: the use of electronic healthcare records with LASSO regression. Oncotarget 2017;8:49637–45. 28548951 10.18632/oncotarget.17870 PMC5564794

[8] Raith EP Udy AA Bailey M. Prognostic accuracy of the SOFA Score, SIRS Criteria, and qSOFA score for inhospital mortality among adults with suspected infection admitted to the intensive care unit. JAMA 2017;317:290–300. 28114553 10.1001/jama.2016.20328

[9] Funk D Sebat F Kumar A. A systems approach to the early recognition and rapid administration of best practice therapy in sepsis and septic shock. Curr Opin Crit Care 2009;15:301–7. 19561493 10.1097/MCC.0b013e32832e3825

[10] McLymont N Glover GW. Scoring systems for the characterization of sepsis and associated outcomes. Ann Transl Med 2016;4:527–1527. 28149888 10.21037/atm.2016.12.53 PMC5233540

[11] Mitchell M Levy AR Phillips GS. Surviving sepsis campaign: association between performance metrics and outcomes in a 7.5-year study. Crit Care Med 2014;40:1623–33. 10.1007/s00134-014-3496-0 25270221

[12] Vallet B. Bench-to-bedside review: endothelial cell dysfunction in severe sepsis: a role in organ dysfunction? Crit Care 2003;7:130–8. 12720559 10.1186/cc1864 PMC270612

[13] Sandrine K Yann B Anne DA. Endothelium-dependent vasodilation in the skin microcirculation of patients with septic shock. Shock 2003;19:274–80. 12630529 10.1097/00024382-200303000-00013

[14] Davis JS Yeo TW Thomas JH. Sepsis-associated microvascular dysfunction measured by peripheral arterial tonometry: an observational study. Crit Care 2009;13:R155. 19778457 10.1186/cc8055 PMC2784378

[15] Ciro C Katalin M Gábor O. Endothelial dysfunction is a potential contributor to multiple organ failure and mortality in aged mice subjected to septic shock: preclinical studies in a murine model of cecal ligation and puncture. Crit Care 2014;18:511. 25223540 10.1186/s13054-014-0511-3 PMC4177582

[16] Duffy MJ Mullan BA Craig TR. Impaired endothelium-dependent vasodilatation is a novel predictor of mortality in intensive care. Crit Care Med 2011;39:629–35. 21242802 10.1097/CCM.0b013e318206bc4a

[17] Motro B Itin A Sachs L. Pattern of interleukin 6 gene expression in vivo suggests a role for this cytokine in angiogenesis. Proc Natl Acad Sci USA 1990;87:3092–6. 1691500 10.1073/pnas.87.8.3092 PMC53840

[18] Colotta F Sciacca FL Sironi M. Expression of monocyte chemotactic protein-1 by monocytes and endothelial cells exposed to thrombin. Am J Pathol 1994;144:975–85. 8178946 PMC1887349

[19] Pierrakos C Vincent J-L. Sepsis biomarkers: a review. Crit Care 2010;14:R15. 20144219 10.1186/cc8872 PMC2875530

[20] Inge G Andrei P Anca R. Biomarkers of inflammation and the etiology of sepsis. Biochem Soc Trans 2020;48:01–14. 10.1042/BST20190029 32049312

[21] Zonneveld R Martinelli R Shapiro NI. Soluble adhesion molecules as markers for sepsis and the potential pathophysiological discrepancy in neonates, children and adults. Crit Care 2014;18:204. 24602331 10.1186/cc13733 PMC4014977

[22] Amalakuhan B Habib SA Mangat M. Endothelial adhesion molecules and multiple organ failure in patients with severe sepsis. Cytokine 2016;88:267–73. 27701021 10.1016/j.cyto.2016.08.028 PMC5121929

[23] Hao Ou, Xianzhong Xiao, Yu Jiang, et al. Expression of microRNA-23b in patients with sepsis and its effect on leukocytes and the expression of E-selectin and ICAM-1. *Exp Ther Med*. 16:4707–4711. 10.3892/etm.2018.6759 PMC6257422 30542424

[24] Susana G-O Mikel A Iratxe Seijas. Identification of a panel of serum protein markers in early stage of sepsis and its validation in a cohort of patients. J Microbiol Immunol Infect 2018;51:465–72. 28655573 10.1016/j.jmii.2016.12.002

[25] Laramee M Chabot C Cloutier M. The scaffolding adapter Gab1 mediates vascular endothelial growth factor signaling and is required for endothelial cell migration and capillary formation. J Biol Chem 2007;282:7758–69. 17178724 10.1074/jbc.M611327200

[26] Carmeliet P Moons L Luttun A. Synergism between vascular endothelial growth factor and placental growth factor contributes to angiogenesis and plasma extravasation in pathological conditions. Nat Med 2001;7:575–83. 11329059 10.1038/87904

[27] Shalaby F Rossant J Yamaguchi TP. Failure of blood-island formation and vasculogenesis in Flk-1-deficient mice. Nature 1995;376:62–6. 7596435 10.1038/376062a0

[28] Gerber HP McMurtrey A Kowalski J. Vascular endothelial growth factor regulates endothelial cell survival through the phosphatidylinositol 39- kinase/Akt signal transduction pathway. Requirement for Flk-1/KDR activation. J Biol Chem 1998;273:30336–43. 9804796 10.1074/jbc.273.46.30336

[29] Bates DO. Vascular endothelial growth factors and vascular permeability. Cardiovasc Res 2010;87:262–71. 20400620 10.1093/cvr/cvq105 PMC2895541

[30] Aslan A van Meurs M Moser J. Organ-specific differences in endothelial permeability-regulating molecular responses in mouse and human Sepsis. Shock 2017;48:69–77. 28151770 10.1097/SHK.0000000000000841 PMC5457831

[31] Peng Y Gao M Jiang Y. Angiogenesis inhibitor endostatin protects mice with sepsis from multiple organ dysfunction syndrome. Shock 2015;44:357–64. 26125086 10.1097/SHK.0000000000000427

[32] Sugioka K Mishima H Kodama A. Regulatory mechanism of collagen degradation by keratocytes and corneal inflammation: the role of uro-kinase-type plasminogen activator. Cornea 2016;35: (Suppl 1): S59–64. 27661072 10.1097/ICO.0000000000000995

[33] Dinesh P Rasool M. uPA/uPAR signaling in rheumatoid arthritis: shedding light on its mechanism of action. Pharmacol Res 2018;134:31–9. 29859810 10.1016/j.phrs.2018.05.016

[34] Lian S Xia Y Nguyen TT. Docosahexaenoic acid inhibits tumor promoter-induced urokinase-type plasminogen activator receptor by suppressing PKCdelta- and MAPKs-mediated pathways in ECV304 human endothelial cells. PLoS One 2016;11:e0163395. 27654969 10.1371/journal.pone.0163395 PMC5031411

[35] Jin T Bokarewa M Tarkowski A. Urokinase-type plasminogen activator, an endogenous antibiotic. J Infect Dis 2005;192:429–37. 15995956 10.1086/431600

[36] Mondino A Blasi F. uPA and uPAR in fibrinolysis, immunity and pathology. Trends Immunol 2004;25:450–5. 15275645 10.1016/j.it.2004.06.004

[37] Yang K-Y Liu K-T Chen Y-C. Plasma soluble vascular endothelial growth factor receptor-1 levels predict outcomes of pneumonia-related septic shock patients: a prospective observational study. Crit Care 2011;15:R11. 21219633 10.1186/cc9412 PMC3222041

