# Supplementary material for: Diagnosis of sepsis with inflammatory biomarkers, cytokines, endothelial functional markers from SIRS patients
Source: Medicine (Baltimore). 2022 Feb 18;101(7):e28681. doi: 10.1097/MD.0000000000028681 (PMC9281918; doi:10.1097/MD.0000000000028681)
Supplement: Supplemental Digital Content [file medi-101-e28681-s001.doc]

<0.001

0.01

<0.001

0.00 <0.001

4

0.01

8

CD62E/E-Selection

(ng/ml)

CD62P/P-Selection

(ng/ml)

A) B) C)

200 150 80

150 60

100

100 40

50

50 20

0

Control SIRS Sepsis

0

Control SIRS Sepsis

0

Control SIRS Sepsis

D) E) F)

4 50

600

400

200

0

Control SIRS Sepsis

3

2

1

0

Control SIRS Sepsis

40

30

20

10

0

Control SIRS Sepsis

<0.001

<0.001

VEGF IL-1 β (pg/ml)

(pg/ml)

MFG-E8 (ng/ml)

TFPI (ng/ml)
